# Mechanisms Linking Excess Adiposity and Carcinogenesis Promotion

**DOI:** 10.3389/fendo.2014.00065

**Published:** 2014-05-01

**Authors:** Ana I. Pérez-Hernández, Victoria Catalán, Javier Gómez-Ambrosi, Amaia Rodríguez, Gema Frühbeck

**Affiliations:** ^1^Metabolic Research Laboratory, Clínica Universidad de Navarra, Pamplona, Spain; ^2^Centro de Investigación Biomédica en Red de Fisiopatología de la Obesidad y Nutrición, Instituto de Salud Carlos III, Madrid, Spain; ^3^Department of Endocrinology and Nutrition, Clínica Universidad de Navarra, Pamplona, Spain

**Keywords:** adipose tissue, carcinogenesis, obesity, inflammation, adipokine, hypoxia, insulin resistance

## Abstract

Obesity constitutes one of the most important metabolic diseases being associated to insulin resistance development and increased cardiovascular risk. Association between obesity and cancer has also been well established for several tumor types, such as breast cancer in post-menopausal women, colorectal, and prostate cancer. Cancer is the first death cause in developed countries and the second one in developing countries, with high incidence rates around the world. Furthermore, it has been estimated that 15–20% of all cancer deaths may be attributable to obesity. Tumor growth is regulated by interactions between tumor cells and their tissue microenvironment. In this sense, obesity may lead to cancer development through dysfunctional adipose tissue and altered signaling pathways. In this review, three main pathways relating obesity and cancer development are examined: (i) inflammatory changes leading to macrophage polarization and altered adipokine profile; (ii) insulin resistance development; and (iii) adipose tissue hypoxia. Since obesity and cancer present a high prevalence, the association between these conditions is of great public health significance and studies showing mechanisms by which obesity lead to cancer development and progression are needed to improve prevention and management of these diseases.

## Introduction

Obesity has reached epidemic proportions constituting one of the most important diseases of this century (1). Despite body mass index (BMI) being used for a long time as the best way to diagnose and define obesity, recent studies have proposed that BMI misclassified subjects with increased cardiovascular risk factors (2). This finding highlights that BMI underestimates the real prevalence of overweight and obesity, since it only considers weight and height of the patients and not total body fat, which really defines the obesity-associated comorbidity risk. In this regard, other indicators to determine the prevalence of obesity have been proposed with body fat percentage being one of the most useful due to its superior ability to stratify patients according to their metabolic and cardiovascular risks (3, 4).

Health care expenditure in severe obesity (BMI ≥ 40 or ≥35 kg/m^2^ with manifest serious comorbidities) is greater than the investment in any other medical condition (5). This is not only due to direct consequences of obesity, but also due to their associated comorbidities. There are numerous evidences of the relationship between obesity and type 2 diabetes mellitus (T2D) (6), non-alcoholic fatty liver disease (NAFLD) (7, 8) and other chronic liver diseases such as cirrhosis (9), obstructive sleep apnea (OSA) syndrome (10), other respiratory alterations (11) as well as cardiovascular diseases (CVD) including cerebrovascular disorders and hypertension (12–14). Furthermore, evidence about the relationship between obesity and several types of cancer is increasing (15–19). These tumors include colon, breast, endometrium, prostate, thyroid, renal, esophagus, gastric cardia, pancreas, gallbladder, and liver cancer as well as non-Hodgkin lymphoma and multiple myeloma (15, 20–23). Nowadays, cancer is probably one of the most important diseases due to its high prevalence and mortality rates (24). According to the National Cancer Institute, a total of 1,660,290 new cancer cases and 580,350 cancer deaths would take place in the United States in 2013 (25). Increasing trends in incidence of the most common cancers in Europe are also of concern, in particular for colorectal cancer, where incidence is high and still continues to increase (26, 27). A large proportion of the studied cancers are potentially avoidable (28), and several programs to prevent or detect early stages of cancer have been proposed, such as gynecologic or colon cancer population screenings (29–31). Importantly, it has been estimated that 15–20% of all cancer deaths in the United States can be attributed to overweight and obesity (32–34). Obesity and cancer affect millions of people with important consequences; thereby a comprehensive approximation to the mechanisms underlying their relationship is needed to ensure better prevention and treatment strategies.

Higher BMIs have been associated with increased risk of disease while at the same time they have been related with a lower mortality and a better outcome in several chronic diseases (35). This paradoxical finding is shared over a variety of cardiovascular, pulmonary, and renal diseases and is known as the *obesity paradox* (36). There is increasing evidence that patients, especially elderly, with elevated BMI and several chronic diseases exhibit lower all-cause mortality compared with normal-weight patients (37). However, it should be highlighted that the BMI is an anthropometric marker that does not consider total body fat mass and distribution, nutritional status, or other factors influencing health risks (2, 3, 38).

## Relationship between Obesity and Cancer

Large prospective studies show a significant association between excess adiposity and several cancers. There is sufficient evidence relating obesity and tumor development of the colon, breast (post-menopausal women), and prostate among others (18).

Breast cancer is the most frequently diagnosed cancer globally and the second cause of cancer death in women. It has been estimated that breast cancer constituted 30% of all new cancer cases in women in 2013 (25). Overweight and obesity are important risk factors for breast cancer development and, in contrast to other risk factors, such as menstruation age, familiar antecedents or aging, both overweight and obesity are preventable. It has been shown that post-menopausal obese women present a threefold increase in the risk of breast cancer compared to the non-obese control group (39). Moreover, other studies have revealed that higher BMI is associated with worse response to neoadjuvant chemotherapy and worse overall survival (40). Other anthropometric measurements, such as the waist-to-hip ratio, also correlate with breast cancer death risk (41). Worse prognosis or survival rates in obese women with breast cancer have been established in different studies (42–44), showing that obesity is an important risk factor for breast cancer and, therefore, therapeutic measures must be adopted to reduce obesity incidence. It is important to take into account that hormone replacement therapy (45, 46) and mammographic density (47) could be additional confounders in these association studies. The obesity-associated increased risk of post-menopausal breast cancer might be explained by higher rates of peripheral conversion of androgenic precursors to estradiol due to an increased aromatase enzyme activity in adipose tissue (48).

Globally, colorectal cancer remains the third most commonly diagnosed in males and the second in females with a cumulative life risk of developing colorectal cancer of 5% in the general population (49). Although both, the incidence and mortality have been slowly but steadily decreasing, colorectal cancer is the third leading cause of cancer-related deaths (25). The relationship between obesity and colon cancer is well established (50–53). In this regard, it has been estimated that about 30% of all colon cancer cases could be attributable to a BMI higher than 22.5 kg/m^2^ (54). BMI appears to be consistently associated with an increased risk of colorectal cancer in men, but only a weak association in women has been described (48). This gender difference might be explained by sex differences in prevalence and age of the onset of metabolic syndrome, or by a protective effect of estrogens, inducing apoptosis and inhibition of cell proliferation (55, 56). However, a recent meta-analysis showed that BMI is positively related with colon cancer in both men and women, and no gender differences were found (57). In this sense, a positive association between BMI and prevalence of colonic adenoma and advanced polyps has been demonstrated in pre-menopausal women according to hormonal status (58). Obesity has also been associated with worse cancer outcomes, such as recurrence of the primary cancer or increased mortality. Several factors, including reduced sensitivity to antiangiogenic–therapeutic regimens, might explain these differences. The underlying mechanisms linking obesity to colorectal cancer are not completely elucidated, but inflammation, insulin resistance, and a dysregulated adipokine profile are proposed as important factors. Other biological factors such as the gut microbiota (59) or bile acid concentrations are emerging as novel influential factors in obesity-associated tumors (55).

Prostate cancer is the second diagnosed cancer and the sixth cause of cancer-related mortality among men worldwide (60). Because obesity and prostate cancer affect substantial proportions of the male population, the association between these conditions is of great public health significance. In the last decade, multiple epidemiologic studies have suggested that obesity is associated with an increased risk of death from numerous cancer types including prostate cancer (32, 48). Three meta-analyses reported a positive association between obesity and prostate cancer incidence and, although modest, the relative risks were consistent (48, 61, 62). Obesity has also been associated with worse prognostic and malignant transformation of epithelial cells (63, 64). However, there are contradictory studies about the link between obesity and prostate cancer (65) reporting no association (64, 66), and even a protective effect of obesity (67–71). Proposed obesity-associated alterations related to prostate tumor development include lower levels of sex hormone-binding globulin that increase the fraction of biologically available testosterone (15).

Lung cancer is the main cause of cancer-related death in both genders, with an estimated incidence of more than 200,000 cases in 2013 in the United States (25). To date, the relationship between obesity and the incidence of lung cancer remains unclear (72). Controversy still focuses on the effect of smoking in studies investigating the relationship between BMI and lung cancer (73). In case–control studies, odds ratios for lung cancer by levels of BMI showed an increasing linear trend with a lower threshold BMI for current smokers and ex-smokers of both sexes (74, 75). Moreover, an inverse association between BMI and lung cancer has been shown in other studies after adjusting for smoking or waist circumference (76–78). In this sense, a recent meta-analysis indicates that overweight and obesity are protective factors against lung cancer, especially in current and former smokers (72). Recently, the involvement of adipokines in lung cancer is under study due to their emerging carcinogenic and immunomodulatory properties, making them potential mediators of the complex and still unclear multistep carcinogenetic process (79).

Association with obesity has also been proposed for other cancer types, as thyroid, kidney, esophagus, gastric cardia, pancreas, gallbladder, or liver cancer, but further studies are needed to confirm the obesity-attributable risk of tumor development.

## Dysfunctional Adipose Tissue in Obesity and Tumor Development

Obesity is described as an excess of adiposity due to a prolonged status of positive energy balance, which leads not only to changes in adipose tissue distribution but also to metabolic alterations as well as altered cytokine and lipid secretion profiles. Energy balance and fuel homeostasis require a tight equilibrium between energy intake (chemical energy from diet-derived macronutrients) and energy expenditure [basal metabolic rate plus mechanical energy by muscle contractions due to physical activity (PA)] that are regulated by both central and peripheral mechanisms. In this sense, substantial changes have occurred in the patterns of foods consumed with special concern about excess of sugar intake in the diet in many industrialized countries mainly as sugar-sweetened drinks (80, 81).

There are two main types of adipose tissue, white and brown, with different origins and functions (82). The major role of white adipose tissue (WAT) is related to maintaining energy homeostasis by storing triglycerides and releasing fatty acids for energy synthesis. It has been well established that WAT also controls a wide variety of functions including immune and inflammatory regulation, glucose and lipid homeostasis, food intake control, or metabolism by secreting a great number of adipokines (83, 84). WAT is a heterogeneous tissue, consisting of a peripheral subcutaneous component (SAT) and a central intra-abdominal component [visceral adipose tissue (VAT)] (85). Abdominal obesity seems to be of greater pathophysiological concern than subcutaneous fat with an increase in VAT being strongly correlated with the metabolic syndrome and tumor development (86–88). Brown adipose tissue (BAT) was initially described to play a physiological role in animals and infants. In human neonates, BAT is located in specific depots, the interscapular and axillary region, and to a lesser extent, near to the thymus and in the dorsal midline region of the thorax and abdomen (89). However, recent studies using positron emission tomography imaging techniques documented the presence of functional BAT in adult humans (90–92). BAT is specialized in thermogenesis, the production of heat mainly mediated by the uncoupling protein-1 (UCP-1), which produces heat by uncoupling mitochondrial respiration for ATP synthesis (93, 94). Cancer has been associated with cachexia, a complex syndrome that involves profound metabolic imbalances (95). The colorectal tumor-induced cachexia on BAT in mice has been described, finding smaller brown adipocytes with profound delipidation in cachectic tumor-bearing mice (96). Recently, the existence of a third kind of adipose tissue has been proposed, the “beige” or “brite” (brown-like adipocytes) adipose tissue (97). Beige adipose tissue is also a thermogenic adipose tissue influenced by cold-induced signals via the sympathetic nervous system.

Adipose tissue from obese subjects experiments a wide range of modifications due to its expansion and the excessive amount of lipids stored. The limitation in adipose tissue expandability leads to fat accumulation in other parts of the body with lipotoxic consequences (98). Expanded fat depots in obesity are less efficient in storing dietary fatty acids, so that obese subjects exhibit an increase in plasma free fatty acids (FFA) and ectopic fat depots. The higher levels of FFA are susceptible to be substrates for oxidative processes and also may function as mediators of insulin resistance in muscle (99) or liver (100, 101). An analysis of cancer-induced modifications in the lipid profile reveals important clues linking obesity and cancer. Changes in lipid metabolism may promote cancer development by an FFA increase, due to its function as oncogenic signals (18). Reportedly, increased fatty acid synthase (FASN) activity has been shown in breast cancer cell lines (102), ovarian tumors (103), or cancer precursor lesions in different locations (colon, stomach, esophagus, and oral cavity) (104). The inhibition of the FASN activity reduces the cancer cells’ proliferative capacity, suggesting that FFA act as an energy source for cancer cells. In addition, some studies show a relationship between mutations in the FASN enzyme and cancer incidence (105). Furthermore, FASN has been proposed as an important biomarker of overnutrition-induced insulin resistance (106). Elevated FFA levels derived either from the cancer cell or exogenous fat sources may promote a more aggressive tumorigenic phenotype with the implication of monoacylglycerol lipase (107). Finally, different studies suggest that cancer cells may use lipids directly from adipocytes as energy source, promoting tumor growth (108).

## Pathophysiological Mechanisms Relating Visceral Adipose Tissue Excess and Cancer

Several mechanisms whereby obesity can favor cancer development and progression have been proposed, including the obesity-associated low-grade inflammation, endocrine alterations, insulin resistance, and hypoxia–angiogenesis processes.

### Adipose tissue inflammation, a microenvironment favoring tumorigenesis

Obesity is characterized by a chronic state of low-grade inflammation (109). Adipose tissue is composed of mature adipocytes and stromal cells, which include preadipocytes, fibroblasts as well as vascular and immune cells. The interaction between immune cells and adipocytes leads to inflammation and subsequent adipose tissue dysfunction. Chronic inflammation is characterized by a sustained response to diverse stress signals, which leads to adipose tissue remodeling (110–112). This process includes adipocyte hypertrophy, immune cell infiltration, angiogenesis, and fibrosis during the progression of inflammation (113–116) accompanied with changes in the adipokine production profile (117). The link between chronic inflammation and cancer development was first noticed over 100 years ago by Virchow, when he observed an abundance of leukocytes in neoplastic tissue (118). Since then, the role of chronic inflammation as a precursor of cancer development has been observed in multiple cancer types (119). In animal models of human cancers, inflammation has been shown to influence tumor promotion and progression (120–122). Like adipose tissue, tumor microenvironment is composed of multiple cell types including epithelial cells, fibroblasts, mast cells, and cells of the innate and adaptive immune system that favors a pro-inflammatory and pro-tumorigenic environment (123–127).

#### Macrophages, obesity, and cancer

Growing evidence reveals that adipose tissue from obese subjects is markedly infiltrated by macrophages that participate in inflammatory pathways with important roles in obesity-associated comorbidities (128). Adipose tissue macrophages (ATM) enhance the levels of inflammatory markers dysregulated in cancer, suggesting that macrophages from peritumoral adipose tissue are locally involved in promoting carcinogenesis (129–131). The presence of tumor-associated macrophages (TAM) also contributes to the pro-inflammatory tumor environment. The recruitment of TAM to the tumor microenvironment is largely dependent on the monocyte chemoattractant protein-1 (MCP-1). In this regard, levels of MCP-1 in tumor tissue have been highly correlated with the accumulation of TAM in ovarian, breast, and pancreatic cancer (123, 132).

**Figure 1:**
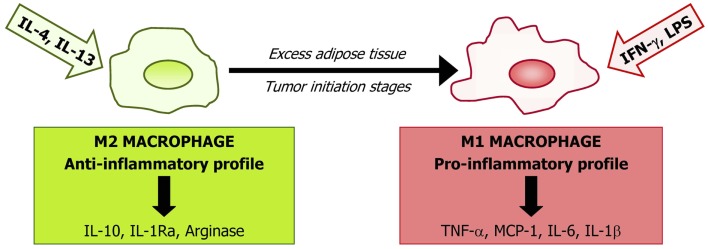
**Macrophages are representative of the innate immune system and represent a large proportion of the stromovascular cell fraction in adipose tissue**. The phenotype of macrophages depends on the subset of monocytes upon arrival at target tissues being probably determined by the local microenvironment. Based on their cytokine profile secretion and cell surface markers, macrophages are classified into two main types: the “classical” macrophages named M1 in contrast to the “alternatively activated” M2. M1 macrophages are the first line of defense against intracellular pathogens and are classically stimulated by interferon (IFN)-γ or by lipopolysaccharide (LPS). M1 induce the secretion of inflammatory cytokines [interleukin (IL)-1, IL-6, tumor necrosis factor (TNF)-α, or monocyte chemoattractant protein (MCP)-1]. Alternative activation, resulting from induction by the Th2 cytokines interleukin IL-4 and IL-13, is associated with tissue repair and humoral immunity producing immunosuppressive factors, including IL-10, IL-1Ra, and arginase. Obesity and initial tumor stages induce a phenotypic switch from an anti-inflammatory M2-polarized state to a pro-inflammatory M1 state.

Local environment factors are able to determine polarization of macrophages through two general states: M1 (pro-inflammatory) and M2 (anti-inflammatory) macrophages (Figure 1). Activation of M1 macrophages is mediated mainly through the nuclear factor κ-light-chain-enhancer of activated B cells (NF-κB) and Jun N-terminal kinase (JNK)/activator protein-1 (AP-1) system. M1 macrophage-derived tumor necrosis factor (TNF)-α and interleukin (IL)-18, 12, and 23 have been identified as important mediators in several chronic inflammatory diseases including obesity and cancer (133–136). Excess of M1 macrophage polarization is related to insulin resistance in obese subjects (137) with M1 macrophages being also differentiated in the tumor initiation stages. Obesity could lead to tumor initiation by the denominated extrinsic pathway, in which tumorigenesis is attributable to a chronic inflammatory condition through the NF-κB pathway (138). NF-κB activation in tumor cells promotes carcinogenesis by increasing their aggressive potential by triggering autocrine growth factor cascades and by inhibiting proliferation control mechanisms, including apoptotic signals (139). NF-κB involvement in tumorigenesis has been shown in various tumor models (140). M1 macrophages also contribute to tumor development by TNF-α and IL-6 signaling, enhancing carcinogenesis by increasing cell proliferation and neoangiogenic cell properties (141, 142). In addition, TNF-α production by macrophages activates the Wnt/β-catenin pathway, which is also associated with tumor development by a non-resulting anti-inflammatory response (143, 144). M2 macrophage activation is mainly mediated by the IL-4-induced activation of Janus kinase (JAK)/signal transducer and activator of transcription (STAT) transduction signaling. It has been shown that IL-10 secreted by M2 macrophages improves insulin signaling, with a protective role in obesity-induced insulin resistance (145). Moreover, several factors are involved in M2 activation, such as peroxisome proliferator-activated receptor (PPAR)-γ, Krüppel-like factor 4, AMP-activated protein kinase (AMPK), or sirtuin-1 (SIRT1) (146).

In adipose tissue from obese subjects, there is a phenotype switch from M2 to M1 macrophages leading to an increase in general inflammatory markers (147). Moreover, during tumor progression, an M2 to M1 transition of ATM has been shown. However, in later tumor stages, a trophic and immunomodulatory M2-like adipose tissue macrophage phenotype is recruited to the tumor environment. It has been proposed that M2 differentiation could act as a compensatory mechanism, trying to re-establish a homeostatic environment in a pathological condition (144). Mechanisms involved in this M1–M2 switch are not well understood, but molecular pathways related to cyclooxygenase (COX)-2, toll-like receptor (TLR), or Notch signaling have been proposed to play an important role (148–150). Macrophage switch from M1 to M2 in tumor setting occurs via many different receptors, signaling pathways, and transcription factors as a result of the coexistence of pro- and anti-inflammatory signals present during tumor progression in the tumor environment.

#### Dysregulated adipokine profile in obesity and cancer

The progression of tumors toward malignancy requires the interaction of various cytokines, growth factors, and transcription factors. Adipose tissue acts as an important endocrine organ via the synthesis of several adipokines, which regulate insulin sensitivity, lipolysis, control of energy intake, or inflammatory processes (151–153). Obesity affects the secretory profile of adipokines leading to alteration in multiple physiological processes (154–164). Adipokines are linked to tumor development and progression due to their plentiful actions on different cell types, mainly by exerting their effects through inflammatory pathways. Adiponectin and leptin are two of the most important adipocyte-specific adipokines.

##### Adiponectin

Adiponectin, also known as Acrp30, ADIPOQ, apM1, or GBP28, is a 30-kDa protein mainly secreted by adipocytes (165). Adiponectin presents a carboxy-terminal globular domain and an amino-terminal collagen domain that forms characteristic multimers (166). Adiponectin exists as full-length adiponectin or as a globular fragment. Globular adiponectin is generated by proteolytic cleavage of full-length adiponectin, but it circulates in very small amounts in the bloodstream (167). Globular adiponectin exists as a trimer, whereas full-length adiponectin can be found as a low-molecular weight (trimer), middle-molecular weight (hexamers), and high-molecular weight (12–18mers) with different biological functions (168, 169). Both globular and full-length adiponectin promote an increased fatty acid oxidation and glucose uptake in the liver and skeletal muscle as well as a decreased hepatic gluconeogenesis. These biological effects are mediated by two transmembrane adiponectin receptors, AdipoR1 and AdipoR2, which are expressed in many tissues and activate the downstream targets AMPK, PPAR-α, and p38 MAPK (167).

Several studies suggest that hypoadiponectinemia or reduced concentrations of adiponectin and PPAR-α in obesity may be one of the mechanisms linking obesity and cancer development as well as progression. Importantly, adiponectin levels are decreased in obesity-associated insulin resistance (170) and cancer (171). A negative correlation between adiponectin levels and the risk of developing colorectal (52, 172, 173), endometrium (174), breast (175), prostate (176), or pancreas cancer (177) has been shown. In animal models, a protective role for adiponectin by suppressing colorectal cancer development has been suggested (178). *Adipoq*-knockout mice showed a significant higher polyp formation compared with wild-type mice under a high-fat diet. Furthermore, it was also demonstrated that *Adipor1*, but not *Adipor2*-deficient mice show an increase in colonic epithelial cell proliferation, suggesting that adiponectin suppresses this biological process under a high-fat diet (178). Reduced adiponectin levels in the obese state lead to the development of insulin resistance and compensatory chronic hyperinsulinemia. Increased insulin levels lead to reduced liver synthesis and secretion of insulin-like growth factor-binding protein (IGFBP)-1 and -2, resulting in increased levels of bioavailable insulin-like growth factor (IGF)-1. Insulin and IGF-1 promote cellular proliferation and inhibit apoptosis in many tissue types, leading to carcinogenesis (15, 171). Low adiponectin levels are potentially associated with carcinogenesis, indirectly through its effects on TNF-α and tumor cell proliferation and directly by its effects on the regulation of hematopoiesis and system, selective binding to several mitogenic growth factors, and inhibition of NF-κB (171, 179). The adiponectin protective effects in tumors also include the inhibition of leptin proliferative signaling and inducing cell apoptosis (180).

##### Leptin

Leptin, the *OB* gene product, acts as a key mediator in body weight regulation (181–184). In adipose tissue, the secretion levels of leptin are strictly controlled and maintain a balance to ensure adequate regulation of food intake and energy expenditure under physiological conditions. Contrary to adiponectin, leptin levels are increased in obese individuals (185). Reportedly, treatment with leptin promotes cell growth, inhibits apoptosis, and modulates migration of cancer cells (186). In this regard, an overexpression of leptin receptors in various cancers like breast (187) or colon (188) has been also shown. Tumor cells from papillary thyroid cancer also show increased expression levels of the leptin receptor being associated with a more aggressive phenotype (189). Mature adipocytes secrete both adiponectin and leptin with preadipocytes showing a primarily secretion of high leptin levels (190). An increase of the preadipocyte pool in obese subjects is related to an increase in leptin levels, with proangiogenic and promitogenic properties (191). At the same time, high leptin levels attract more inflammatory cells and promote monocyte to macrophage differentiation, maintaining the obesity-associated state of chronic inflammation. In summary, leptin has an important role in the development of a large variety of malignancies, predominantly acting through the JAK/STAT pathway, which modulates phosphatidylinositol 3-kinase (PI3K)/Akt and extracellular signal-regulated kinase (ERK) 1/2 signaling pathways increasing the expression of anti-apoptotic proteins (XIAP), inflammatory markers (TNF-α, IL-6), angiogenic factors [vascular endothelial growth factor (VEGF)], and also the hypoxia-inducible factor-1α (HIF-1α). These processes promote cancer cell survival, proliferation, and migration.

##### Tumor necrosis factor-α

TNF-α is a pro-inflammatory cytokine secreted by adipocytes with increased secretion levels in obese subjects (192). TNF-α was first identified as a macrophage-derived factor that induces the necrosis of tumor cells, but when its antitumoral activity was tested on cancer patients, a paradoxical tumor-promoting role became apparent (193–195). At present, the pro-inflammatory role of TNF-α has been linked to all steps involved in tumorigenesis, including cellular transformation, survival, proliferation, invasion, angiogenesis, and metastasis (195, 196). Animal models have shown a positive relationship between TNF-α and tumor development and progression in liver (197) and colorectal cancer (195) with elevated circulating concentrations in different tumoral types (198, 199). In addition, TNF-α is not only produced by a wide variety of tumor cells (200) but also by adipocytes. Levels of TNF-α are increased in obesity, indicating a role for this cytokine in the obesity-associated inflammation and particularly in insulin resistance and diabetes.

##### Interleukin-6

Interleukin-6 is another major pro-inflammatory cytokine secreted by adipose tissue, which shows increased levels in obese subjects. It has been implicated in inflammation-associated carcinogenesis (201, 202). IL-6 modulates the expression of genes involved in proliferation, survival, and angiogenesis via the JAK/STAT signaling pathway (203). A relationship between IL-6 and carcinogenesis has been shown for renal cell carcinoma (204), gastric cancer (205), or colorectal cancer, among others (200, 206). Moreover, elevated levels of IL-6 in cancer patients correlate with disease aggressiveness and poor prognosis (207, 208). Other studies have revealed the role of IL-6 as an anti-inflammatory cytokine acting via the classic signaling through the activation of the IL-6 receptor on the cellular membrane of specific cell types such as macrophages, neutrophils, some T-cells, and hepatocytes (209, 210).

##### C-reactive protein

C-reactive protein (CRP) is an acute-phase reactant protein induced as a response to inflammatory conditions and different cytokines including IL-6. Elevated levels of CRP in adipose tissue and serum of obese subjects have been shown (211) as well as in patients with a variety of malignancies (212). Prospective studies have shown a higher risk of developing cancer in those with elevated serum CRP (213, 214). Elevated CRP is a significant predictor of lower survival rates in patients with several cancers, including esophageal, colorectal, hepatocellular, pancreatic, urinary bladder, renal, ovarian, and cervical cancer, after surgical resection. CRP can serve as an additional prognostic predictor for survival and post-treatment monitoring in cancer patients (215).

##### Resistin

Resistin, also known as Fizz3, is a 12-kDa cysteine-rich protein that plays a role in the development of insulin resistance and obesity in rodents (216). While adipose tissue constitutes the main source of resistin in mice, this molecule is secreted mainly by monocytes and macrophages in humans (217). Human resistin is implicated in the pathogenesis of atherosclerosis by promoting smooth muscle cell proliferation and migration, ICAM-1 and VCAM-1 expression, as well as via regulating patterns of adhesion and inflammation in atherosclerotic plaques (218, 219). Several colorectal cancer studies have shown a positive correlation between resistin and tumor size based on T-staging and tumor grading (172, 220). Resistin has also been postulated as a target molecule, which associates with clinicopathological features and prognosis of pancreatic ductal adenocarcinoma (221). Resistin has been postulated as a pro-inflammatory adipokine (222), exhibiting significantly higher serum levels in patients affected by breast cancer in comparison to controls. Additionally, resistin has been proposed as a regulator of the human choriocarcinoma cell invasive behavior and endothelial cell angiogenic processes (223).

##### Monocyte chemoattractant protein-1

MCP-1 is a chemokine implicated in the infiltration of macrophages in adipose tissue. Higher levels of MCP-1 can be found in obese subjects compared with lean subjects (224). The presence of infiltrating macrophages as a consequence of increased MCP-1 levels has been shown to correlate with cancer metastasis and poor prognosis in a variety of human carcinomas (172, 225). MCP-1 overexpression has been reported in both ovarian cancer (226, 227) and colorectal cancer (228).

##### Visfatin

Visfatin also known as pre-B cell colony enhancing factor (PBEF) or nicotinamide phosphoribosyltransferase (NAMPT) is a 52-kDa protein with apparently insulin-mimetic actions (229) and increased plasma concentrations in patients with obesity and T2D (230). Since several authors could not replicate the insulin-mimetic activities of visfatin as well as its ability to bind the insulin receptor, Fukuhara and colleagues were forced to retract some of their original findings (231). However, visfatin stimulates glucose-induced insulin secretion in β-pancreatic cells (232, 233). Visfatin has been further identified in inflammatory cells with its levels being reportedly increased in various inflammatory conditions (229). Visfatin has been related with carcinogenesis and tumor progression, as well as with chemotherapy response (234) in colorectal (235, 236) and breast cancer (237).

##### Osteopontin

Osteopontin (OPN), also known as secreted phosphoprotein-1 (SPP1) and bone sialoprotein-1, among others, is a phosphoprotein expressed by a wide variety of cell types, including adipocytes (238). The expression levels of OPN are increased in obesity (160, 239) as well as in other pathophysiological processes including neoplastic transformation, progression of metastases, and promotion of cell survival (240). OPN is also expressed in many cancers, with elevated circulating levels and tissular tumor expression being associated with poor prognosis in gastric and liver cancers (241, 242).

##### Chitinase-3-like protein-1

Chitinase-3-like protein-1 (CHI3L1), also known as YKL-40 or human cartilage glucoprotein-39, is a member of mammalian chitinase-like proteins with increased gene expression levels in obesity and T2D (155, 243). Recently, YKL-40 has been proposed as a new inflammatory marker related to insulin resistance (244). YKL-40 is also increased in several types of solid tumors contributing not only to the development of an inflammatory state but also to cell proliferation, inhibition of apoptosis, stimulation of angiogenesis, and regulation of extracellular tissue remodeling (245). Increased YKL-40 expression has been detected in glioblastoma multiforme (246), papillary thyroid carcinoma (247), extracellular myxoid chondrosarcoma (248), colon cancer (131), and diverse cancer cell lines, being suggested as a useful cancer prognostic biomarker (249).

#### Consequences of oxidative stress

Reactive oxygen species (ROS) comprise hydrogen peroxide (H_2_O_2_), superoxide anion (^·^O_2_), and hydroxyl radical (^·^HO) and induce DNA mutations contributing to cancer development and progression. Potential sources for ROS production in mitochondria include xanthine oxidase, cytochrome P450 oxidases, uncoupled nitric oxide synthases, and NADPH oxidases (250).

Reactive oxygen species exert a wide range of effects in cancer cells depending on their concentrations. In general, low levels of ROS are mitogenic and promote cell proliferation and survival, while intermediate levels cause transient or permanent cell cycle arrest and induce cell differentiation (251). At high levels, ROS can easily react with membrane lipids (causing membrane permeability alteration), DNA (inducing damage and genomic instability), and proteins (promoting oxidative modifications that result in less active enzymes or proteins more susceptible to proteolytic degradation). These facts suggest a possible protective effect of high local ROS levels in cancer progression (252). However, although ROS production does not irreversibly alter cell viability, they can act as a primary messenger, modulating several intracellular signaling cascades leading to cancer progression. Indeed, it has been demonstrated that ROS activate the MAPK, PI3K/Akt, phospholipase C-γ1 (PLCγ1), protein kinase C, NF-κB, and JAK/STAT pathways (253). The increase of fat mass in obesity has been correlated with the increase of markers of systemic oxidative stress in both human and mice (254, 255). In this regard, an increase in the NADPH oxidase subunits in obesity has been described (254), suggesting that adipose tissue may constitute a source of ROS, releasing them into the peripheral blood, and affecting the function of remote organs (256). Adipose tissue from lean subjects expresses antioxidant enzymes (catalase, superoxide dismutase-1, and glutathione peroxidase) for managing ROS production, with the expression of these antioxidant enzymes downregulated in adipose tissue from obese individuals (254, 255, 257, 258).

Therapeutic approaches to cancer involving ROS metabolism have been proposed in recent years. Experimental results have proposed that the ROS increase is involved in apoptosis induction by chemotherapeutic anticancer agents (259). Cancer cells with increased oxidative stress are likely to be more vulnerable to damage by further ROS insults induced by exogenous agents (260). Therefore, manipulating ROS levels by redox modulation constitutes a way to selectively induce cancer cell death without causing significant toxicity to normal cells (261).

### Hormonal links in obesity-associated cancers

A substantial number of cancers are linked to a hormonal etiology such as tumors of the breast, endometrium, ovary, prostate, testis, and thyroid. Adipose tissue excess is thought to be related with these types of tumors due to the abnormal levels of several hormones encountered in obesity, most notably increased estrogen levels (262, 263). Estrogen is a known growth factor with different biosynthesis between pre- and post-menopausal women (264). Pre-menopausal women mainly synthesize estrogens in the ovary, however, after the menopause peripheral sites including adipose tissue, are the primary source of estrogens. In this regard, for post-menopausal women, significant increases in estrone, estradiol, and free estradiol are associated with increasing BMI (262). Estrogen binds to at least three major classes of receptors, ER-α, ER-β, and GPR30 (265) activating numerous growth-promoting genes, including growth factors and growth-enhancing protooncogenes such as c-fos and c-myc (266–268). The primary mediator of post-menopausal estrogen biosynthesis is aromatase, which is found in adipose tissue as well as tumor tissue itself (269). In the expanding adipose tissue, this mechanism of production leads to a local increase in estrogen levels favoring tumor development (15). Different meta-analysis indicated a higher risk of breast cancer in post-menopausal women with high serum estrogen levels (270, 271). However, the role of blood levels of estrogen in obesity-associated colon cancer is controversial. Evidence showed a worse marked effect of obesity in men than women in the carcinogenesis of colon cancer, pointing to a protective effect of estrogens, which are increased in obesity (56).

In addition to excess estrogen, hyperplasia and carcinogenesis may also stem from a decrease in circulating progesterone levels. In obese women, lack of progesterone due to anovulation, similar to that observed in the polycystic ovarian syndrome, may contribute to endometrial cancer risk (272).

### Obesity-associated insulin resistance

Insulin is a key hormone involved in the control of glucose and lipid homeostasis with insulin actions being mediated by a transmembrane insulin receptor, a heterotetrameric glycoprotein consisting of two α-subunits and two β-subunits linked by disulfide bonds (273). Insulin promotes cellular response through many pathways, including PI3K/Akt signaling leading to AMPK activation. AMPK has been implicated in regulating cellular functions including energy state, fuel metabolism, mitochondrial biogenesis, protein and ceramide synthesis, as well as cell growth and proliferation (274). In addition, its activation was initially shown to inhibit TNF-α-induced inflammation, insulin resistance, apoptosis, and oxidative stress. AMPK activity is diminished in adipose tissue from severely obese subjects who are insulin resistant compared to equally obese individuals who are insulin sensitive (275). Studies evaluating insulin secretion, as reflected by C-peptide levels, have pointed out a correlation between hyperinsulinemia and poor clinical outcome and death in prostate cancer (276). A recent study has shown that aldo–keto reductase 1B10, which plays a critical role in tumor development and progression through promoting lipogenesis and eliminating cytotoxic carbonyls, is induced by insulin through the activator protein-1 (AP-1) signaling pathway in human hepatocellular carcinoma cells (277). Most recent reports have also suggested that insulin has mitogenic and anti-apoptotic effects in endometrial cancer and that the activation of insulin receptors and Akt is associated with more aggressive features (278, 279).

Since insulin also acts as a growth factor, there are some molecules, called insulin-like peptides (ILPs), which can bind to the insulin receptor and trigger intracellular responses similar to those triggered by insulin. In humans, the most important ILPs are IGF-1 and IGF-2. The mitogenic and anti-apoptotic environment caused by elevated levels of insulin and IGF-1 in obesity accelerates the stepwise accumulation of mutations and, hence, favor carcinogenesis (280). Studies evaluating IGF-1 circulating concentrations and cancer risk show important effects on cancer development and progression. Circulating IGF-1 binds mainly to the major IGF-binding protein, IGFBP-3 (281). Results of early studies evaluating the risk of prostate (282), breast (283), colorectal (284), and lung cancer (285) suggest that high circulating IGF-1 concentrations are associated with an increased risk, whereas high IGFBP-3 concentrations are associated with a lower risk (286, 287). Associations between IGF-1, IGFBP-3, and cancer risk vary by cancer site. Circulating concentrations of IGF-1 increase the risk of non-smoking-related malignant diseases (288). Other ILPs, like IGF-2, have also been proposed to have a relationship with carcinogenesis (279).

### Hypoxia, angiogenesis, and cancer development

Adipose tissue presents an adequate exposure to nutrients and oxygen due to the extensive capillary network surrounding each adipocyte. However, there is substantial evidence that hypoxia develops in adipose tissue as the tissue mass expands, and the reduction in O_2_ concentrations is considered to underlie the obesity-associated inflammatory response (289). Cancer may be promoted by the hypoxic and angiogenic environment of obese adipose tissue, in conjunction with the described elevated circulating levels of cytokines existing in obese subjects (290).

Studies in hepatocellular carcinoma have shown a higher accumulation of ATM in tumor regions poorly vascularized (291, 292). Under low oxygen conditions, both tumor cells and macrophages establish a proangiogenic program mediated by HIF-1, which is a transcriptional activator complex constituted with two types of subunits, an inducible α subunit (HIF-1α, HIF-2α, or HIF-3α), and the constitutively expressed HIF-1β subunit. Hypoxia stabilizes HIF-1α, preventing its post-translational hydroxylation and consequently proteasome-mediated degradation. In addition, hypoxia promotes HIF-1α association with HIF-1β, as well as cofactor recruitment (293). HIF-1α is also transcriptionally regulated by NF-κB (294). ATM adaptation to hypoxia is mediated by the induction of HIF-1 and HIF-2-regulated genes, including VEGF, fibroblast growth factor (FGF)-β, and IL-8, as well as glycolytic enzymes (295). Furthermore, the HIF-1 pathway has been demonstrated to play a role in macrophage recruitment and activation. As suggested for NF-κB and STAT-3, HIF-1 might be a potential target in hepatocellular carcinoma therapy (296). HIF-1 has been also proposed as a key molecule implicated in tumor metastasis (297, 298) and a possible metabolic target in cancer therapy (299–301). Inflammatory molecules like TNF-α are also secreted by adipose tissue in response to hypoxia and macrophage–monocyte infiltration (280).

## Therapeutic Strategies

Since obesity is a result of a prolonged positive energy balance, caloric restriction and PA are key strategies to reduce BMI, fat mass, and improve metabolic abnormalities. Although pharmaceutical approaches for obesity and T2D are used, the main pillar of treatment is lifestyle changes. In this regard, it has been recently reported that behaviors concordant with the Nutrition and Physical Activity Cancer Prevention Guidelines are associated with lower risk of total, breast, and colorectal cancers and lower cancer-specific mortality in post-menopausal women (302).

### Physical activity

Sedentary behavior and insufficient PA are strongly related with obesity and metabolic syndrome development. Animal studies have shown metabolic beneficial effects of aerobic exercise training independent of dietary changes (303). However, it has been demonstrated that the combination of PA and dietary intervention is more effective than only an increase in PA (304). There are evidences of benefits of physical exercise to normalize obesity-altered lipid patterns (305, 306), improve glucose metabolism (307), change the growth hormone/IGF-1 axis (308), and consequently reduce the risk of obesity-associated comorbidities. Studies in humans have also shown a direct relationship between BMI, PA, and mortality with cancer (309). In a large cohort study of middle-age women, both the excess of weight (considered as a BMI ≥ 25 kg/m^2^) and sedentary habits were significantly associated with an increased mortality. It has been estimated that excess weight and physical inactivity could account for 31% of all premature deaths and 21% of deaths from cancer among non-smoking women (310). A great variety of studies has reported the advantages of PA in several types of cancer, such as breast (311, 312), colon (313, 314), or prostate cancer (315).

### Diet

Both epidemiological studies and experiments in animals suggest that alterations in caloric intake or in the quality of the diet may significantly influence the risk of cancer development and progression. A higher risk of cancer development related to diets rich in calories, overabundant in alcohol and animal fats, and/or deficient in vegetal products has been reported. By contrast, cancer risk was lower in diets with a higher intake of fruit and vegetables. High-fiber cereal consumption has also been linked to a reduced risk of colorectal cancer (16, 316). Recently, the role of ω-3 polyunsaturated fatty acids in cancer prevention has been highlighted (317, 318). These fatty acids exert their effects by targeting different stages of cancer development, including cell proliferation and survival, inflammation, or angiogenesis (319, 320).

A cell-cycle regulation by nutrients has been shown in studies on *Saccharomyces cerevisiae*. Growth experiments under glucose-limited conditions suggested that coupling of circadian, metabolic, and cell division cycles is essential for genome integrity (321). However, the coupling of these cycles is highly dependent on the experimental conditions and can be uncoupled under other nutrient-limiting conditions (322). The relationship between nutrient availability and genome alterations could play a role in obesity-related cancer due to an abnormal energy state in obese subjects. Therefore, aerobic glycolysis or the Warburg effect links the high rate of glucose fermentation to cancer (323). Moreover, sugar intake and consumption of sweet cakes and cookies have been associated with increased risk of endometrial cancer (324). Biological mechanisms whereby high-sugar foods consumption might increase risk of cancer development are related to the development of hyperglycemia, stimulation of insulin production, and insulin resistance (325, 326).

Caloric restriction could be a strategy to prevent cancer by reducing changes induced by obesity and general inflammatory profile (327). In this regard, studies in prostate cancer and murine prostate cancer models suggest that caloric restriction and weight loss may reduce the risk of prostate cancer-specific mortality (71). Positive effects of weight loss and PA have also been demonstrated in colorectal cancer (55).

### Bariatric surgery

When PA and dietary changes are not enough to reduce BMI, bariatric surgery is a useful strategy to reduce excess of fat mass and prevent weight gain in morbid obesity. There are different surgical techniques that combine variable degrees of restriction and malabsorption. Beneficial effects of bariatric surgery in reducing obesity-associated alterations have been largely described. A clinical improvement in patients with T2D after bariatric surgery procedures has been shown (328, 329). Noteworthy, there are studies that suggest a preventive effect of bariatric surgery on diabetes development (330). Positive effects over other comorbidities include OSA (331), dyslipidemia (332), hepatic fibrosis (333), and cardiovascular risk (334, 335). Furthermore, there is evidence showing that bariatric surgery-induced weight loss reduces cancer risk in some retrospective cohort studies (336, 337). The mechanisms that may lead to a reduced cancer incidence in subjects under bariatric surgery include reduction in sex steroid plasma levels; decreased circulating fatty acid, adipokines, and pro-inflammatory cytokines levels; improvement in insulin sensitivity and reduction of fat mass leading to a lower ectopic fat accumulation (338). The Swedish Obese Subjects (SOS) study, a prospective and controlled intervention trial involving 2,010 obese patients who underwent bariatric surgery and 1,037 contemporaneously matched obese subjects, found a significantly lower number of first-time cancers after inclusion in the surgery group (gastric banding, vertical banded gastroplasty, and gastric bypass) than in the control group, but only in women. It has been postulated that since gastric bypass reduces hyperinsulinemia, this could also be a biological mechanism by which bariatric surgery exerts protective effects on cancer incidence (339). Future studies with larger patient numbers and longer follow-up periods are required to firmly establish whether this might also have beneficial effects for men in the long term (340).

## Conclusion

**Figure 2:**
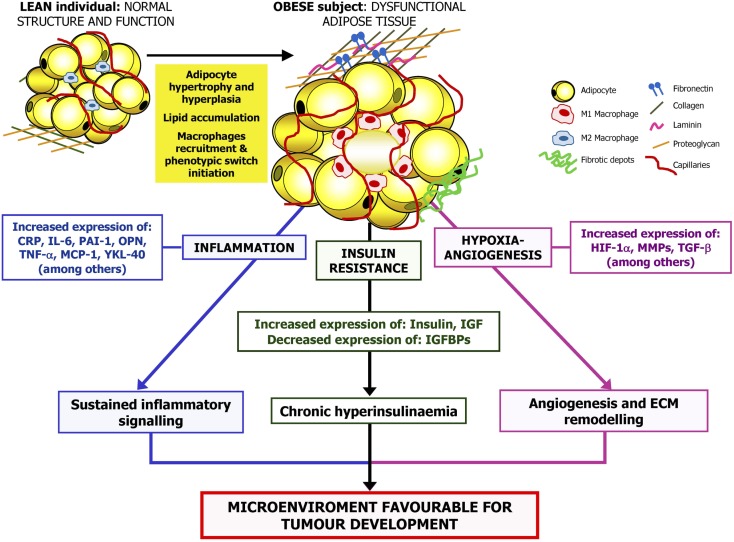
**Proposed mechanisms linking obesity and cancer**. The excess of adipose tissue, especially abdominal obesity, is related to changes in circulating lipid concentrations, reactive oxygen species levels as well as adipokine and hormone secretion profile. Obesity is also linked to adipocyte hypertrophy and hypoxia, aggravating the inflammatory state. Therefore, the adipose tissue-derived inflammatory cytokines, the production of angiogenic factors by adipocytes, or infiltrating M1 macrophages that take place in obesity together with the obesity-associated insulin resistance may promote the stimulation of a microenvironment favorable for tumorigenesis. CRP, C-reactive protein; HIF-1α, hypoxia-inducible factor-1α; IGF, insulin growth factor; IGFBP, insulin-like growth factor-binding protein; IL, interleukin; MCP-1, monocyte chemoattractant protein 1; MMP, matrix metalloproteinase; OPN, osteopontin; PAI-1, plasminogen activator inhibitor-1; TGF-β, transforming growth factor β; TNF-α, tumor necrosis factor α; YKL-40, chitinase-3-like protein.

Obesity is a potentially avoidable high prevalent risk factor for developing different health problems including tumor development. There are increasing evidences relating obesity and cancer, with a well-established relationship between obesity and breast, colorectal, prostate, or lung cancer among others. It has been proposed that an important percentage of all cancer deaths may be attributable to obesity. Due to the high prevalence and repercussion of these conditions, the understanding of mechanisms of obesity-associated tumorigenesis is an important objective in the prevention and treatment of these pathologies. Future interventions to reduce the prevalence of obesity are needed to prevent cancer development in these patients. In this regard, it has been shown that increased PA, healthy dietary habits, and bariatric surgery may be appropriate to reduce cancer risk in obese patients. In addition, a comprehensive approximation to the underlying molecular mechanisms involved in obesity-related carcinogenesis may provide specific targets implicated in pathways by which obesity leads to tumor progression. Reduction of inflammatory signaling, improvement of insulin sensitivity, or counteracting the hypoxia-inducible factors appears to be relevant pathways in prevention of cancer development and reducing its progression (Figure 2).

## Conflict of Interest Statement

The authors declare that the research was conducted in the absence of any commercial or financial relationships that could be construed as a potential conflict of interest.
